# Resurfacing total hip replacement–a therapeutical approach in postmenopausal women with osteoporosis and hip arthrosis

**Published:** 2011-05-25

**Authors:** D Popescu, R Ene, C Cirstoiu

**Affiliations:** Orthopedics–Traumatology Clinic, University Hospital, BucharestRomania

**Keywords:** resurfacing, osteoporosis, arthroplasty, bisphosphonate

## Abstract

**Aim**: Patients with incipient hip arthrosis may benefit from a relatively new therapeutical approach using resurfacing total hip replacement, but in those with associated osteoporosis, this type of surgical intervention is contraindicated, given the poor quality of osteoporotic bones. We assessed the efficacy of the antiosteoporotic pharmacological therapy to improve bone quality and bone strength in postmenopausal women diagnosed with hip arthrosis and osteoporosis thus facilitating the hip surgical intervention.

**Methods**: We evaluated 20 postmenopausal women aged between 53–60 years diagnosed with osteoporosis according to the WHO criteria, by using dual–energy X–ray absorptiometry (DXA) for bone mineral density measurements. All these patients had low hip T score (osteopenia/ osteoporosis) and also incipient hip arthrosis. The surgical approach was delayed for 12 months and all the patients received bisphosphonate therapy with calcium and vitamin D supplements. DXA scans were performed after 12 months of therapy in all the patients.

**Results**: A surgical intervention with resurfacing total hip replacement was performed in 12 of the 16 patients presenting with increasing BMD, 4 of them showing elements of rapidly advancing hip arthrosis to a stage that made this type of intervention impossible. We chose not to use this technique in the group with stable BMD (4 patients). All 12 women surgically treated had a favorable post–operative outcome without experiencing a femoral neck fracture during the surgical intervention or during the twelve–month follow–up. All 20 patients continued to receive bisphosphonate therapy.

**Conclusion**: In postmenopausal women with osteoporosis and associated hip arthrosis, improving bone mass and bone quality with bisphosphonate therapy is necessary and important in order to allow hip arthroplasty, by using the technique of resurfacing, avoiding the risk of intra–operative fractures and  with a favorable  post–operative long–term  outcome.

## Introduction

In the modern sense, osteoporosis is defined as a systemic skeletal disease characterized by low bone mass and deterioration of the bone microarchitecture, resulting in increased bone fragility and predisposition to fracture. Osteoporosis is currently the most frequent metabolic bone disease, a more recent definition correlating this skeletal disease with the alteration of the bone strength, reflecting both bone density, and bone quality.

Significant progress has been made in recent years not only in defining this public health problem, but also in identifying the multiple pathogenic mechanisms underlying the occurrence of osteoporosis, enabling interventional strategies to reduce the morbidity and mortality associated with it, as well as to decrease the significant socio–economic costs involved by osteoporosis in the context of the increase of life expectancy and consequently, the increase of the population at risk for this disease.

The aging process and/or estrogen deficiency remain the main pathophysiological links involved in the loss of bone mass in primary osteoporosis: **involutional** and **postmenopausal**. After the age of 40, as one ages, the loss of bone capital is of approximately 0.5%–1% per year, in women the occurrence of menopause leading to a more accelerated rate of bone loss of 1%–2% per year, especially in the first 5–10 years of menopause. The occurrence of menopause at the bone level has the effect of a substantial increase in the remodeling process which results in a significant negative bone impact, causing loss of bone mass. During postmenopause, the loss of bone mass has two stages: an initial stage characterized by an accelerated–predominant loss of trabecular tissue, followed by a stage during which the loss continues, but at a lower rate and especially at the level of the cortical bone.

The initial stage is recorded in the first 5–10 years after the occurrence of menopause and is materialized in the loss of approximately 20%–30% of the trabecular bone and of only 5%–10% of the cortical bone. During this stage, remodeling takes place mainly at the level of the endosteal surface, thus explaining the loss of the trabecular bone. The evaluation of the biochemical markers of the bone turnover seems to indicate that at the time of the menopause the bone resorption markers increase by 90%, whereas the bone formation markers only increase by 45%.

In the next stage, the loss of bone mass continues, but at a lower rate, being associated with the progressive increase of the seric value of the parathyroid hormone and of the bone turnover markers, the studies suggesting a causal relationship between these two biological changes.

The imaging techniques used for the diagnosis of osteoporosis are based on the measurement of the bone mineral density and of the bone mineral content. Dual–energy X–ray absorptiometry (DXA) is currently the most widely used technique and it is considered the ‘gold standard’ in diagnostic means, enabling not only the diagnosis, but also the monitoring of the loss of bone mass, as well as the effectiveness of the various drug strategies employed. 

## Material and method

In recent years, the number of postmenopausal women with osteoporosis has increased. At the same time, the age at which surgery is indicated in coxarthrosis has decreased, increasingly younger people choosing total hip replacement instead of a conservative treatment. One of the most modern techniques in hip arthroplasty is the resurfacing one, which preserves a large bone capital, which has a lower risk of dislocation caused by wide movement (due to the large head of the prosthesis) and a longer life (due to the lower friction forces given by the metal–on–metal hip replacement). Moreover, the revision of such prosthesis often consists only in the replacement of the femoral component with a classical stem–shaft which should preserve the metal–on–metal hip replacement with a large diameter femoral head. The resurfacing endoprosthesis is a new method, distinctly superior to the endoprosthesis techniques used, from the point of view of the immediate and distance post–operative recovery, followed by the full resumption of the function of the pelvic limb and a minimum rate of postoperative complications. The performances of this prosthesis are obvious in well selected cases (young patients with intense physical activity) after a surgical indication correctly formulated.

**Figure 1:**
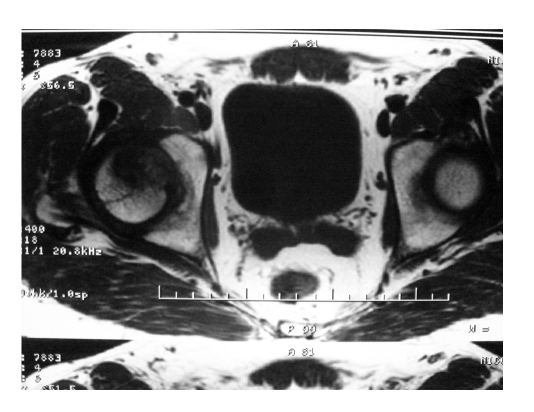
Pelvis MRI

During the period 2005–2010, 22 total hip replacements were performed in the Clinic of Orthopedics and Traumatology of the University Emergency Hospital of Bucharest, in patients younger than 60 with NACF (aseptic femoral head necrosis) primary or secondary coxarthrosis in the initial stage and postmenopausal osteoporosis. The following were used as investigation methods: hip X–rays –two incidents (front and profile), pelvis MRI (Figure 1) and DXA. After setting the diagnosis, the surgical procedure was delayed for six months, during which the patients received a conservative treatment for coxarthrosis and treatment with bisphosphonates to increase BMD. X–ray examinations and DXA at 0.3 and 6 months preoperatively were performed.

## Results

Resurfacing total hip replacements were performed on 14 patients, in whom an increase in BMD over 1.5 at the DXA examinations performed every 0 to 6 months was found and in whom the evolution of hip arthritis was slow. In six patients' treatment with bisphosphonates the bone quality did not improve sufficiently and in two cases of aseptic femoral head necrosis, the disease progressed rapidly and did not allow the implantation of a resurfacing prosthesis.

### Regarding the perioperative planning, the following stages were followed:

**Figure 2:**
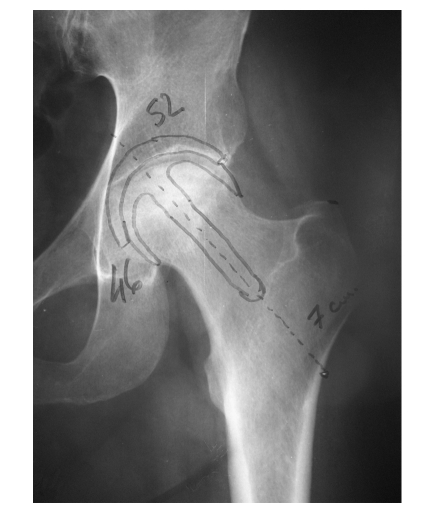
Determination of the size of the implant

As a standard method of investigation a hip X–ray in the anterior–posterior incidence was performed in a 1/1 ratio, on which the preoperative planning was performed to determine the size of the implant (Figure 2).

**Figure 3:**
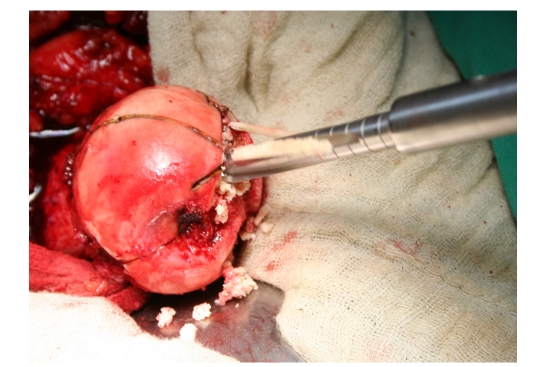
The preparation of the femoral head

The approach was posterolaterally made, with the patient placed in lateral decubitus. After the incision of the external rotator muscles, full capsulotomy was performed. The femoral head was turned and positioned in the anterior–superior part of the cotiloid cavity to facilitate its preparation by milling. As a rule, the milling is performed so as to achieve a diameter smaller than 2 mm, than the acetabular prosthetic component, which has to be implanted. The preparation of the femoral head is performed by using a guidance to center the stem, then for the reversed mills (Figure 3).

Intraoperatively, three factors are taken into consideration when deciding the type of implantation of a resurfacing prosthesis:

- the damage to the femoral head of < 35%
- the normal appearance of the junction between the femoral head and the femoral neck
- sufficient bone capital remaining at the level of the femoral head to ensure the stability of the stem.

**Figure 4:**
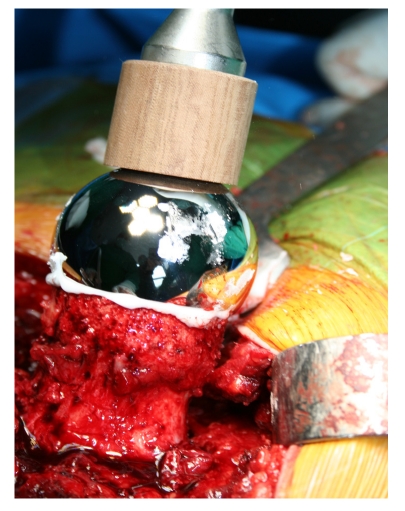
The fixation of the stem is performed by using low–viscosity cement

The fixation of the stem is performed by using low–viscosity cement (Figure 4).

All the patients were administered antithrombotic prophylaxis, with heparin administered subcutaneously, starting with the day of the surgery until the 45th day of post–operative care. 

The immediate postoperative results were good in all cases, the patients having an early motor recovery. The patients were mobilized with progressive loading by means of a walking frame during the first seven days, then were fully loaded by using the help of two crutches for another seven days, giving up one of the crutches before the 21^st^ day. One month postoperatively they were mobilized with full loading without support, the movements being mostly recovered. The recovery was rapid, within a short period of time the patients being able to make complex movements of normal amplitude. They resumed normal activity (work, sports) 6–8 weeks postoperatively.

**Figure 5:**
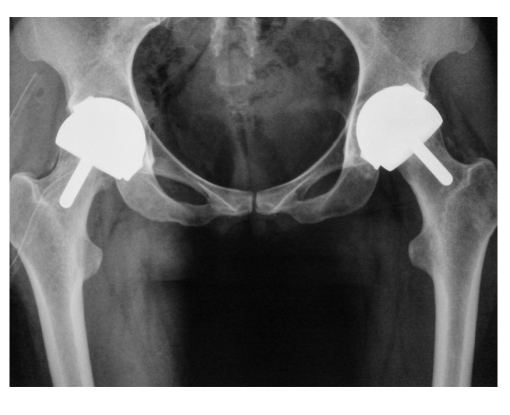
Bilateral total hip replacements

**Figure 6:**
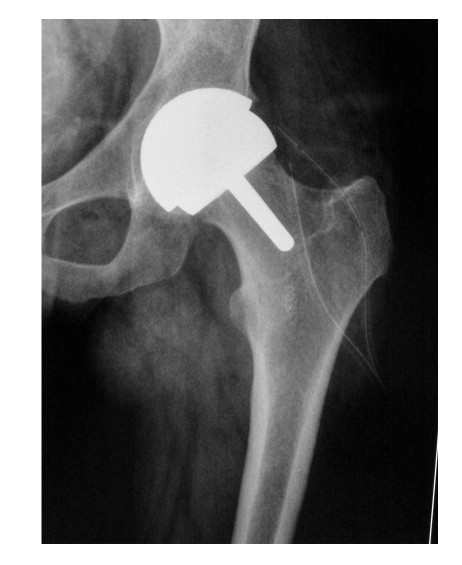
Unilateral total hip replacements

All the patients were followed up clinically and radiologically (Figure 5) at 4 weeks, 6 weeks, 3 months, 6 months, 12 months, then annually.

 No femoral neck fractures were reported, both during surgery and during the first 12 months after surgery, the patients benefitting from all the advantages of a resurfacing prosthesis.

The patients continued to receive the treatment with bisphosphonates for the treatment of osteoporosis.

## Discussions

The use of the resurfacing total hip prosthesis brings real benefits to the patients. Out of all these, the most important are:

- the preservation of the femoral head and femoral canal, thus avoiding the loss of capital bone in the event of a review.
- the large size of the stem significantly reduces the risk of dislocation
- the quick, complete post–operative recovery with the normal motility of the hip joint
- the physiological transmission of the forces at the level of the femoral canal by means of the femoral head and neck
- the metal–on–metal hip replacement avoids the occurrence of the particle disease

## Conclusions

We recommend the delay of the hip arthroplasty in patients with postmenopausal osteoporosis and the administration of the treatment with bisphosphonates to increase BMD for 12 months, in order to have possibility to use a resurfacing prosthesis. Postmenopausal osteoporosis successfully treated is not an absolute contraindication for resurfacing total hip replacement. The introduction of the resurfacing total hip replacement in women with moderate osteoporosis has led to an increased quality of life postoperatively. 
